# French Physicians on the Drink Problem

**Published:** 1896-12

**Authors:** 


### French Physicians on the Drink Problem.

The traditional belief that France is a country of great sobriety, compared with other nations, is not correct. The French Director of Public Instruction has recently given some very startling figures, which are not denied. To find the amount of spirits used in absolute alcohol, a comparison was made with other wine and beer-drinking countries, and it was assumed that all beer contained an average of 3 percent of alcohol, wine 10 percent, and cider 9 percent. Then adding up the consumption of alcohol under the various names of wine, cider, beer, brandy and other spirits, and dividing by the population, it was found that every person in France consumed thirteen quarts of absolute alcohol a year.

Comparing this with other nations, this was found to be the highest rate. Switzerland came next, with ten quarts for each person per annum. Then followed Germany, England, Norway, Sweden, Canada, Italy and Spain. The amount of spirits used has been increasing in France and Belgium, and decreasing in Scandinavia, Canada and Great Britain.

The following appeal has been scattered broadcast by the Blue Cross Society:

"Citizens! Two doctors of Paris, in a recent work, have formulated these sinister conclusions: (1) Alcohol is a poison;

(2) France offers to us the desolating spectacle of a nation literally rushing toward decay through alcohol. Patriots! A revolution develops itself. Do you wish a new tyrant to reign over us? In all parts of the country cafes and taverns are multiplied; it is the multiplication of suicides, of crimes, of insanity. Alcoholism diminishes the birth-rate and increases the death-rate; alcoholism enfeebles, intellectually and morally, the leading classes, while it increases the army of the helpless, the poor, and the discontented; alcoholism costs our country yearly a thousand million francs; alcoholism, therefore, is the ruin of the public health and endangers the public security. Is it not enough, Frenchmen? Let us unite for the suppression of the demand for alcohol. Let each give up personally, radically, courageously, the use of spirituous liquors. Thou, who will go to shed thy blood at the frontier, art thou ready for sacrifices more obscure but more efficacious? Thou, who will renounce all to save France, wilt thou renounce, in order to deliver her, thy small glass, thy appetizer, thy spoonful of brandy? Down with alcohol! That is to say, long live France!"

The question of what can be done has been brought before the Academy of Medicine several times during the past year in very elaborate papers. The Chamber of Deputies has discussed the situation and listened to many plans. The fact most interesting to us is that a large number of eminent medical men have taken up this problem, and are agitating the subject in every possible way. Two societies, which correspond very nearly to our temperance organization, are officered largely by medical men, who are fearless in their discussions and condemnations of the use of alcohol.

Evidently, a great evolution is going on in this old wine-raising and wine-drinking country.—*Cincinnati Lancet-Clinic.*

